# Cell-by-Cell Alignment of Repeated Specular Microscopy Images from the Same Eye

**DOI:** 10.1371/journal.pone.0059261

**Published:** 2013-03-14

**Authors:** Daniel Böhringer, Stefan Lang, Thomas Reinhard

**Affiliations:** University Eye Hospital, Cornea Reading Center, Freiburg, Germany; Glasgow University, United Kingdom

## Abstract

**Purpose:**

Modern specular microscopes (SM) robustly depict the same central area of the corneal endothelium at different time points through a built-in fixation light. However, repeated image acquisitions slightly shift and rotate because of minute changes in head position in the chin and forehead rest. This prevents the manual retrieval of individual corneal endothelial cells (CECs) in repeated measurements because SM images usually lack obvious landmarks. We devised and validated an image registration algorithm that aligns SM images from the same eye to make corresponding CECs coincide.

**Methods:**

We retrospectively selected 27 image pairs for the presence of significant image overlap. Each image pair had been recorded on the same day and of the same eye. We applied our registration method in each image pair. Two observers independently validated, by means of alternation flicker, that the image pairs had been correctly aligned. We also repeatedly applied our registration method on unrelated image pairs by randomly drawing images and making certain that the images did not originate from the same eye. This was done to assess the specifity of our method.

**Results:**

All automated registrations of the same-day and same-eye image pairs were accurate. However, one single image incorrectly failed to trigger the non-match diagnosis twice in 81 registration attempts between unrelated images. As it turned out, this particular image depicted only 73 CECs. The average number of CECs was 253 (range 73–393).

**Conclusion:**

Repeated non-contact SM images can be automatedly aligned so that the corresponding CECs coincide. Any successful alignment can be considered as proof of the retrieval of identical CECs as soon as at least 100 CEC centroids have been identified. We believe our method is the first to robustly confirm endothelial stability in individual eyes.

## Introduction

The corneal endothelial cells (CECs) tightly regulate the hydration of the corneal stroma. Significant cell loss can result in bullous keratopathy, a painful state of corneal edema which usually requires transplantation to restore vision since the CECs do not regenerate sufficiently [1]. CEC preservation is therefore a key safety parameter in many clinical trials involving the anterior segment of the eye [2].

Traditionally, endothelial stability is assessed by means of CEC density [2]. However, it is not currently possible to confirm endothelial stability in individual eyes before and after exposure to a potentially detrimental trial intervention. This is because CEC density estimations are prone to sampling errors [3]. We proposed to eliminate sampling errors by comparing only identical CECs before and after treatment [4]. This would require the alignment of identical CECs which is currently unfeasible because SM images have always shifted and rotated slightly due to variable head positions in the chin and forehead rest [4]. Furthermore, the manual retrieval of identical CECs is virtually impossible because the SM images usually lack obvoius landmarks. We devised and validated an automated image registration algorithm that aligns SM images from the same eye in order to make identical CECs coincide automatically. This is of course only possible when both images overlap to some degree. Fortunately, this image overlap is regulated with a fixation light in all modern SMs. We herein describe our image registration algorithm and the assessment of its sensitivity and specifity in a small trial.

## Results

**Figure 1:**
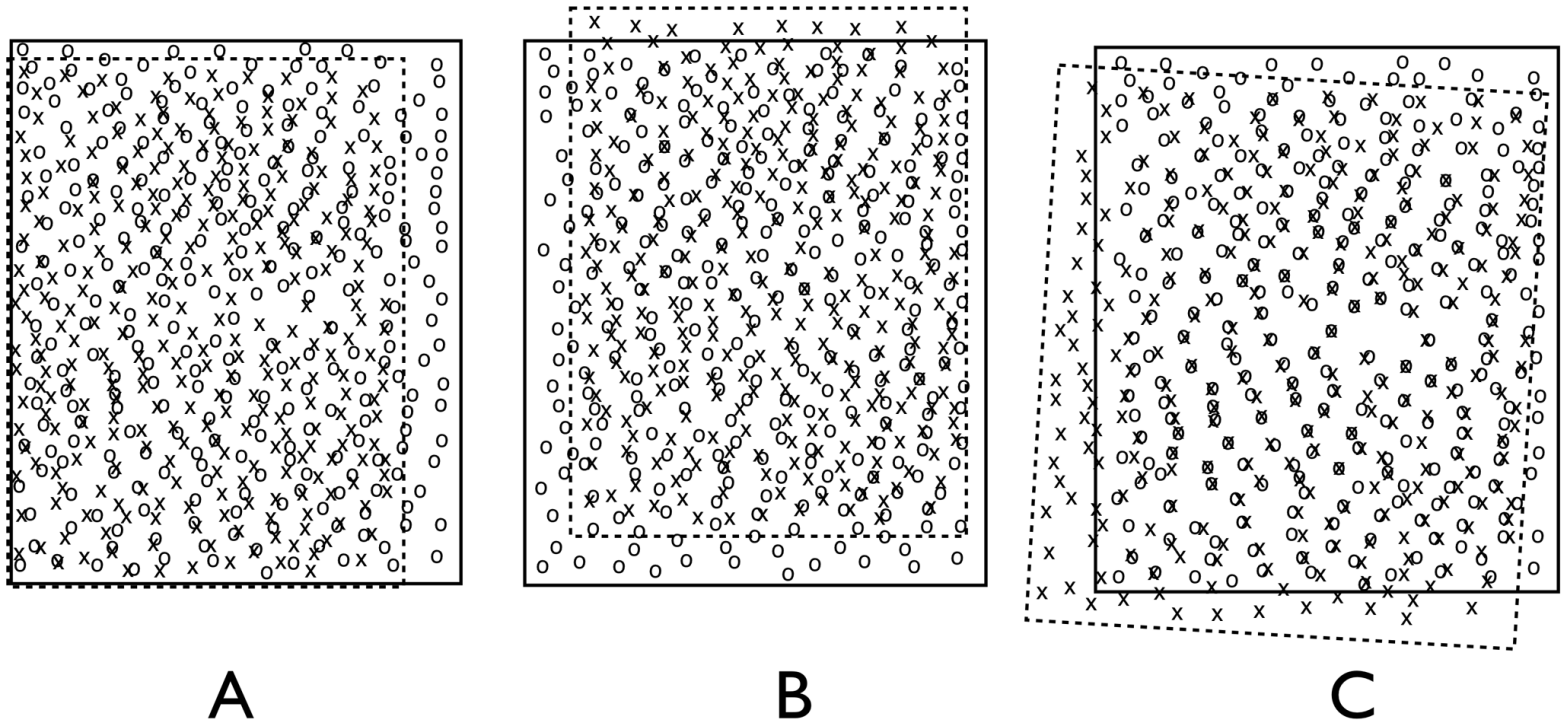
Paradimatic registration result. A: Centroid extraction from the scanned video prints. B: Centroid point sets from image 1 (green) and image 2 (red) after proper alignment. C: Stripe-wise image comparison of image 1 (green stripes) and image 2 (red stripes) after corresponding alignment of image 2. Note that the cell borders are completely continuous between the stripe crossings.

Our automated method consistently identified all overlapping regions in the trial image pairs. This corresponds to a sensitivity of 100%. Two observers independently confirmed the alignments with the help of alternation flicker. Here, the perception of cellular movements during flicker would readily reveal any erroneous alignment. However, all CECs in the overlapping areas remained in place during flicker as judged unanimously by both investigators. Figure 1 depicts the method in a paradigmatic image pair to demonstrate that proper image alignment is actually based on point set registration of the CEC centroids.

We also made 81 registration attempts between randomly-assigned image pairs originating not from the same eye. Here, our method incorrectly failed to report the non-match only twice. As it turned out, the same single image matched incorrectly with two different images originating from another eye. Interestingly, this erroneously-matching image depicted only 73 CECs. This was the image with the lowest number of CECs in our trial. The number of CEC centroids averaged 253 (range 73 to 393). The low number of extracted CEC centroids in this particular image was the result of inhomogeneous illumination and not due to very low CEC density. Furthermore, that image did not display higher degrees of pleomorphism and polymegathism. Closer inspection revealed that our algorithm had extensively scaled and skewed the source image with 73 CECs against substantially larger target images until a complete coincidence was achieved with a subset of the destination CECs. This cannot happen when source and target images are roughly the same size, as is usually the case. However, both erroneous matches would have been readily spotted upon manual review because of grossly unrealistic transformation parameters.

## Discussion

We herein demonstrate for the first time the feasibility of aligning two non-contact SM images in order to coincide the corresponding CECs. We designed our method to assess endothelial stability in clinical trials. In this context, we proposed comparing the baseline SM image to the image taken after exposure to the potentially damaging trial intervention cell-by-cell [4]. If the CECs in both SM images are in fact identical, we can obviously rule out any CEC damage in that area because CECs ultimately shift or enlarge within hours in the proximity of CEC damage [5]. We performed a statistical simulation experiment to assess the power to detect any randomly-distributed CEC loss in the whole cornea based on a sample of the 300 CECs we typically get with our method in the prospective setting. The entire corneal endothelium probably consists of approximately 380000 CECs. This number comes from (arguably simplistic) geometric assumptions of a perfectly spherical cornea, a white to white distance of 11 millimeters and uniform CEC density of 2000 cells per square millimeter. In our simulation, we randomly drew samples from the 380000 CECs a thousand times. We reliably (95% probability) detected a CEC damage percentage as low as 1%. A second sample from a different location would lower the detection threshold to only 0.5% damage. Most importantly, however, we can confidently claim CEC stabiliy in a single patient for the first time.

Cell density comparisons, by contrast, work only in cohorts because they are based on statistical distributions. Statistical comparisons of CEC densities can only prove CEC loss as a matter of principle. If there is no statistically significant CEC loss, we have to assess the statistical power when discussing the chances of CEC stability. To safely rule out (95% probability) a CEC loss of 1% on the basis of a non-significant t-test, a total of at least 5000 patients would have to be analyzed (as calculated with the function under the assumption of a standard deviation of 200 cells per square millimeter). This is of course not feasible when the corneal endothelium is only a safety parameter. For this reason, small degrees of CEC damage based on CEC density comparisons are currently undetectable in many clinical trials. However, an intraocular device that would induce 1% CEC loss over a month may well induce bullous keratopathy within 2 years, depending on the initial CEC density. This has actually happened after anterior chamber lens implantations [6]. One option to slightly alleviate this dilemma would be to take additional SM images at different locations (e.g. superior limbal vs central). However, such data would require sophisticated linear regression models because these additional observations are not fully independent additional samples.

In the present study, we analyzed only same-day measurements, which is why we cannot address how this method performs in case of actual CEC loss. However, our method is robust against smaller degrees of CEC loss, as evident from figure 1B. Here, several centroids are not perfectly superimposed [e.g. at coordinates 410,140, we see two green dots around a red one (the result of imperfect centroid extraction rather than real CEC loss, as evident from figure 1C)]. Actually, our method has indeed been applied to post-keratoplasty eyes, e.g. limbo-keratoplasty [4] and Descemet Membrane Endothelial Keratoplasty (unpublished observation). In both instances, the corresponding CECs were retrieved after 11 months and one month, respectively. If our method fails to match two SM images despite careful attempts to fixate the fixation light and sufficient image quality, we can assume thorough remodelling of the mosaic after substantial CEC loss. However, this would be substantiated by a considerable drop in CEC density.

The manual comparison of two SM images is a strenous and error-prone procedure because SM images typically lack obvious landmarks. We have implemented an algorithm that makes use of the inconspicuous variations in size of the CECs to solve this problem. If cell sizes could be measured perfectly, then a patch of only 15 differently-sized CECs would occur only once in instances for combinatory reasons. Interestingly, even 73 CECs turned out insufficient to achieve perfect specificity in our method. This was, however, the result of “overfitting” that can only happen when a small image is matched against a larger one. This error is easily spotted upon manual review on the basis of a grossly irrealistic projection. According to our small validation study, any successful alignment can be considered as proof of the retrieval of identical CECs, given that a minimum of roughly 100 CEC centroids had been extracted and the image projection withstands manual review.

In summary, we developed a computer program that operates on two grayscale SM images without manual input. Our software either emits the stacked and aligned images, or reports a non-match diagnosis. While we consider our software quite mature, there is still much room for improvement from the image-acquisition perspective. If we could move the fixation light in steps slightly smaller than the image frame width, we could “stich” the images together and thus depict a wide field of the central corneal endothelium. This would allow for higher degrees of certainty when diagnosing endothelial stability in future clinical trials. Further applications of our algorithm could be image quality enhancement and surgical eye tracking. Image quality could be enhanced through repeated image acquisitions and averaging after proper alignment. This could even be done transparently within the SM microscope. Surgical eye tracking based on the endothelial mosaic would be independent of the iris and limbus: both structures are potentially altered during surgery, and are thus a poor source of landmarks for general-purpose surgical eye tracking.

## Materials and Methods

### Image registration algorithm

As the endothelial mosaic can be reconstructed from CEC centroids [7], they provide perfect landmarks for SM image registration. Furthermore, centroids can be automatically extracted from SM images with minimal error [8]. When the centroids are aligned to match eatch other, the underlying images can be aligned with exactly the same transformation. The result of this projection is a properly-aligned image pair for manual cell-by-cell comparisons, which can be done via alternation flicker (see below).

**Figure 2:**
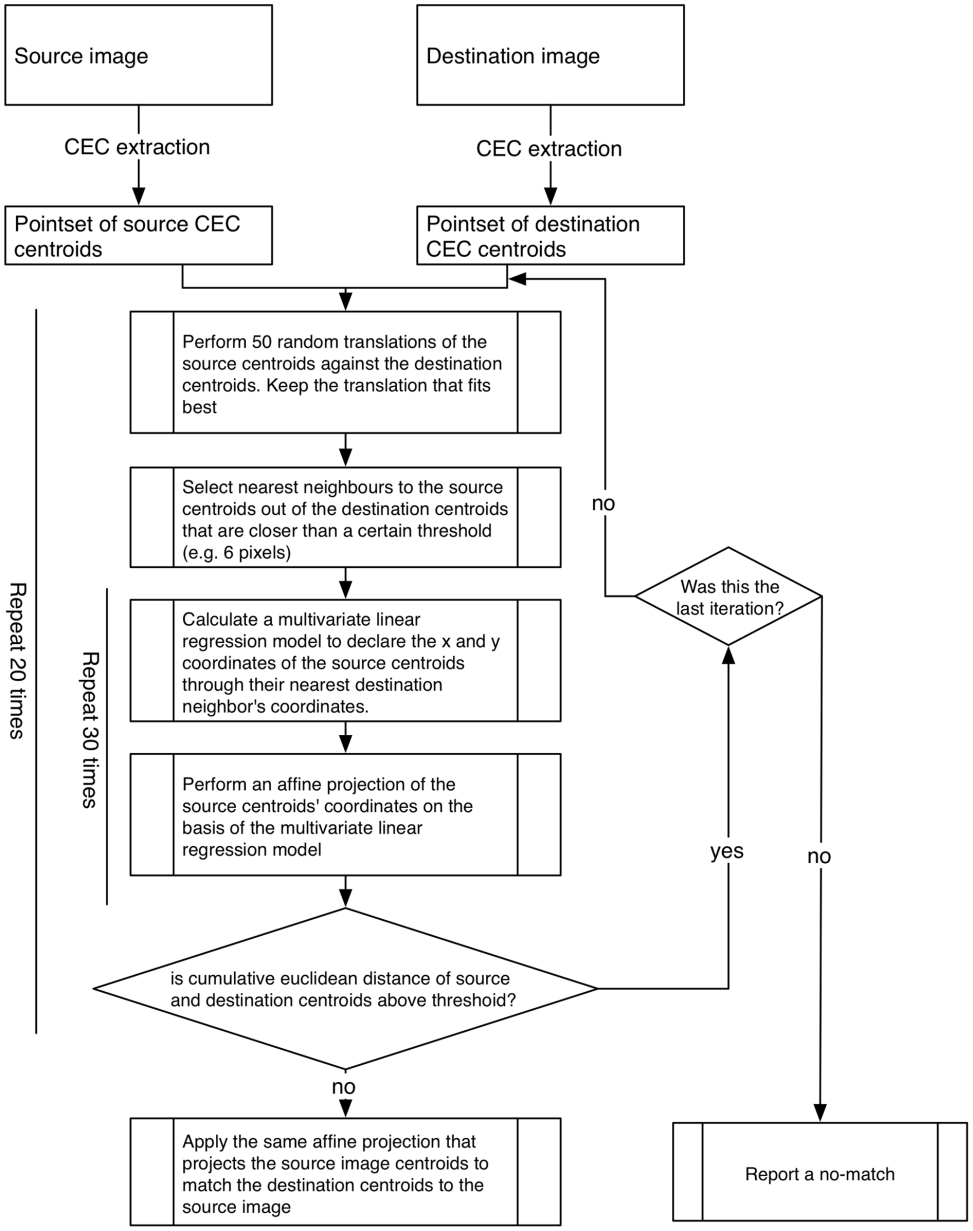
Flow chart of our algorithm. We iteratively align source and destination centroids as closely as possible. We start by performing only translations. We then pair source and destination centroids according to the nearest neighbor principle. These pairs are multivariately correlated through a linear regression model. We affinely transform the destination image pointset with the help of parameters from this regression model. These steps are iteratively repeated in a nested fashion.

**Figure 3:**
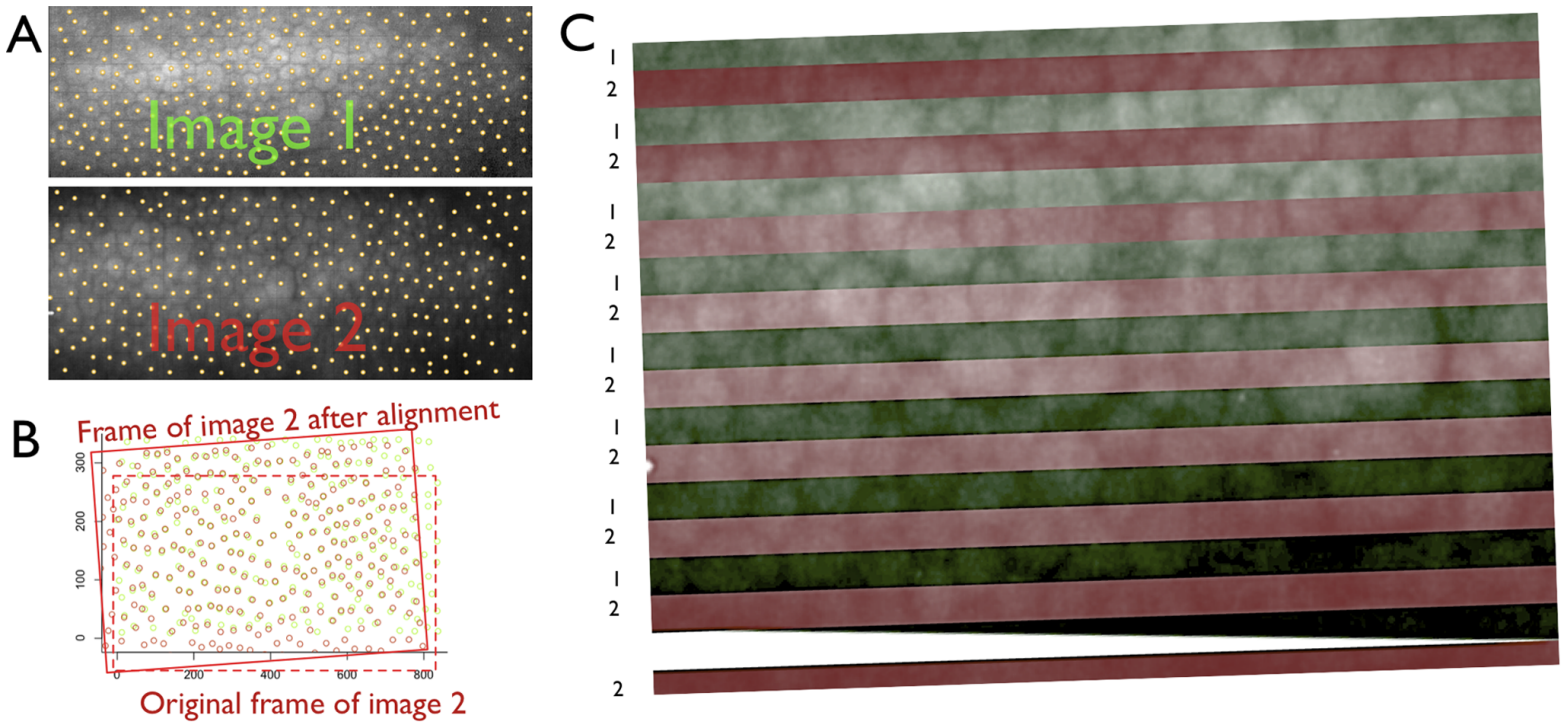
The two chained steps of our pointset registration method. A: Superimposition of the CEC centroids as extracted from the source image (o-shaped) and the destination image (x-shaped). B: Superposition of source and destination CEC centroids after translation of the destination image for maximum correspondence. C: Superimposition of source and destination CEC centroids after additional scaling and rotation.

Our point-set registration algorithm is outlined in Fig. 2. We repeatedly transform the source-image centroids until the maximum number of centroids overlaps with the destination-image centroids. This happens iteratively in two nested loops. Briefly, we align source and destination centroids as closely as possible by translation only (Fig. 3b). Thereafter, we pair corresponding (nearest neighbor) centroids of the source and destination pointsets, respectively. These paired CECs’ coordinates are eventually multivariately correlated through a linear regression model. We affinely transform the destination centroid pointset based on the parameters from this model (Fig. 3c). These procedures are iteratively repeated. In summary, our approach is somehow related to the random sample consensus (RANSAC) principle [9]. If no convergence is reached after all scheduled iterations, the algorithm reports a no-match diagnosis. Our procedure is implemented in the R programming language. On modern hardware, a single matching run takes approximately 60 seconds.

### Validation study

We retrospectively selected 27 image pairs for the presence of significant image overlap. These images had been recorded on the same day. We obtained written informed consent from the participants. The ethics committee of the Albert-Ludwigs-University of Freiburg expressed a favorable opinion and approved our study. The underlying dataset has been described elsewhere [10]: briefly, we acquired multiple SM images in corneal outpatients and healthy volunteers with two different Topcon SP-3000P microscopes. The repeated recordings had been taken within a 10-minute timeframe. All images were printed with video printers. Using custom image analysis software, we digitized these prints and extracted the CEC centroids. The average number of CEC centroids was 253 (range 73–393). The differences in CEC numbers on the image resulted from differences in cell density but also on the extent of inhomogeneous illumination or insufficient image quality.

We applied our registration method to each image pair, each comprising two images from the same eye and same day. Two observers independently validated these automated registrations by means of alternation flicker. The images were presented on modern 27” LCD displays. The images spanned a diagonal size of approximately 20 cm and were viewed at a distance of 40 to 60 centimeters. Alternation flicker is a proven and tested means of detecting glaucomatous changes in the optic disc [11] or brain tumor progression [12]. We also repeatedly applied our registration method on unrelated image pairs. Here, we randomly drew images and made sure that the images did not originate from the same eye. This was done to assess the specifity of our method.
